# Barefoot therapists: barriers and facilitators to delivering maternal mental health care through peer volunteers in Pakistan: a qualitative study

**DOI:** 10.1186/s13033-016-0055-9

**Published:** 2016-03-15

**Authors:** Najia Atif, Karina Lovell, Nusrat Husain, Siham Sikander, Vikram Patel, Atif Rahman

**Affiliations:** Human Development Research Foundation, Islamabad, Pakistan; School of Nursing, Midwifery and Social Work, University of Manchester, Manchester, UK; Institute of Brain, Behaviour and Mental Health, University of Manchester, Manchester, UK; Sangath Centre, Porvorim, Goa, India; The London School of Hygiene and Tropical Medicine, London, UK; Institute of Psychology, Health and Society, University of Liverpool, Liverpool, UK

**Keywords:** Perinatal depression, Task shifting, Peer volunteers, Psychosocial intervention, Thinking Healthy Programme, Low and middle income countries

## Abstract

**Background:**

Perinatal depression is a public health problem in low and middle income countries. Although effective psychosocial interventions exist, a major limitation to their scale up is the scarcity of mental health professionals. The aim of this study was to explore the facilitators and barriers to the acceptability of peer volunteers (PVs)—volunteer lay women from the community with shared socio-demographic and life experiences with the target population—as delivery agents of a psychosocial intervention for perinatal depression in a rural area of Pakistan.

**Methods:**

This qualitative study was embedded in the pilot phase of a larger peer-delivered mental health programme. Forty nine participants were included: depressed mothers (n = 21), PVs (n = 8), primary health care staff (n = 5), husbands (n = 5) and mothers-in-law (n = 10). Data were collected through in-depth interviews and focus groups and analysed using the Framework Analysis approach.

**Results:**

The PVs were accepted as delivery agents by all key stakeholders. Facilitators included the PVs’ personal attributes such as being local, trustworthy, empathetic, and having similar experiences of motherhood. The perceived usefulness and cultural appropriateness of the intervention and linkages with the primary health care (PHC) system was vital to their legitimacy and credibility. The PVs’ motivation was important, and factors influencing this were: appropriate selection; effective training and supervision; community endorsement of their role, and appropriate incentivisation. Barriers included women’s lack of autonomy, certain cultural beliefs, stigma associated with depression, lack of some mothers’ engagement and resistance from some families.

**Conclusion:**

PVs are a potential human resource for the delivery of a psychosocial intervention for perinatal depression in this rural area of Pakistan. The use of such delivery agents could be considered for other under-resourced settings globally.

## Background

Globally, in women of child-bearing age, depression accounts for the largest proportion of the burden associated with mental or neurological disorders [1]. The rates of perinatal depression in lower and middle income countries (LMICs) range from 15.6 % during pregnancy to 19.8 % in the postpartum period [2]. In Pakistan, these rates are even higher than most LMICs, reported to range between 25 and 38 % [3, 4]. In addition to the economic and human costs of maternal depression, children of depressed mothers are at risk of health, developmental, and behavioral problems [5]. Studies from Pakistan have found that infants of depressed mothers have low birth weight [6, 7], lower rates of exclusive breastfeeding [8], higher rates of diarrhoea per year [9], growth retardation [10] and delayed cognitive and motor development [11].

Over 67 % of Pakistan’s population resides in rural areas [12], where only out-patient services are available through PHC facilities, which include Rural Health Centres, Basic Health Units and government dispensaries. These facilities are proportionately few and generally provide no maternal mental health care [13]. Specialist mental health services in rural areas are almost non-existent [12]. The ratio of psychiatrists to patients (1:41,000) is low and scarcity of mental health facilities compounded with lack of mental health training among primary health care (PHC) staff has resulted in a large treatment gap for mental illnesses [14].

In such resource constrained settings ‘task shifting’ has shown to be a potentially effective and feasible approach to address health workforce shortage [15, 16]. It involves shifting delivery of the task from professionals to health workers with fewer qualifications or creating a new workforce with specific training for a particular task [17]. Recent systematic reviews have indicated effective use of task-shifting for the delivery of psychosocial interventions for the prevention and treatment of perinatal depression through community health workers [18, 19]. In Pakistan, a cognitive behaviour therapy (CBT) based Thinking Healthy Programme (THP) was delivered via lady health workers (LHWs)—PHC employee working in the community on mother and child health agendas—and produced significant results favouring the treatment arm [20]. THP has been adopted by the World Health Organization as a low intensity psychological intervention for perinatal depression [21].

However, a major barrier to the scale-up of THP is the burden of work of LHWs. They are the front-line work-force for communicable diseases as well as provision of basic PHC, and their heavy work-load often leaves them little capacity to deliver mental health care as part of their routine work [22]. Thus it is necessary to examine the potential role of other human resources, such as peers. THP has been adapted in Pakistan to make it deliverable through peers (the Thinking Healthy Programme peer delivered (THPP)) and is currently being evaluated for through a cluster randomised control trial.

The term peer has been used in the context of healthcare for those who have common socio-demographic characteristics as the target population and/or use their own experience of overcoming an illness to help others [23–25]. Peers have been successfully used in the delivery of maternal mental health care, but most studies were conducted in high-income countries and focus on quantitative outcomes [24, 26, 27]. Systematic reviews from HICs on peer support indicated promising results, in terms of increased empowerment, confidence, self-esteem, hope, social support and functioning, for both the peers and persons with mental illness [25, 28]. A few experimental studies from LMICs have shown positive mental health outcomes from peer-delivered interventions [29–33]. Singla’s formative study in Goa, India and Rawalpindi, Pakistan showed contextual differences such as levels of urbanisation, education and emancipation of women, can influence stakeholders’ viewpoint about an ideal peer, and the peers’ own expectations from this role [34].

To our knowledge no studies in LMIC have been conducted exploring in depth stakeholders’ experiences of a peer volunteer-delivered psychosocial intervention in a rural, close-knit community. This study was embedded within the pilot phase of the THPP (described below) and was aimed to explore barriers and facilitators to the acceptability of peer volunteers (PVs), prior to implementation of programme utilising this resource.

## Methods

### Settings and participants

The study was conducted in 2013–2014 in the rural sub-district of Rawalpindi, located in the province of Punjab, Pakistan. The relevant socio-demographic information is given in Table 1.

**Table 1:** Sociodemographic of Pakistan

	Variable	Value
1	Population	182 million
2	Life expectancy	65 years
3	Fertility rate	3.8
4	Women in child-bearing age (15–49 years)	27.5 %
5	Population below the poverty line	21 %
6	Overall literacy rate	57 %
7	Women’s literacy rate	34 %
7	Contraception usage	35.4 %
8	Births attended by skilled birth attendant	52.1 %
9	Infant mortality rate	6.9 %
10	Child mortality rate	8.9 %
11	Maternal mortality rates	0.276 %
12	Children underweight	31.5 %

Source

1: THE World Bank. 2013. Data [Online]. Available: http://data.worldbank.org. [Accessed 15 November 2014]

2–3: United Nation Development Report. 2013. Data [Online]. Available: http://www.pk.undp.org/content/pakistan/en/home/countryinfo/. [Accessed 10 October 2014]

4–7: Pakistan Social and Living Standards Measurement Survey. 2013. Pakistan Bureau of Statistics, Government of Pakistan

8–12: Pakistan Millennium Development goals report. 2013. Planning Commission, Government of Pakistan

The data was collected from the two union councils Banda and Jatha Hathiyal with a combined population of about 70,000. Each union council has a Basic Health Unit, delivering PHC services. The economy is primarily agrarian-based, but about a third of people, mostly men, are engaged in non-farm jobs, such as working as unskilled or semi-skilled labourers and self or government employers or serving the army. Majority of families in the study area live in a joint family system (consisting of multiple generations; parents, their children, and the children’s spouses and offspring, living in the same house), and women are generally economically and socially dependent on significant members of their families (husband, mother-in-law, own parents).

The study was embedded within the pilot phase of THPP and included all participants taking part in the pilot study (Table 2). Participants for the pilot study were selected through purposive sampling and included PVs, mothers experiencing perinatal depression, their significant family members and local PHC staff. PVs were lay women from the community who shared similar socio-demographic characteristics and life experiences with the target population. They had an education of at least 10 years, and were able to work in the community. Eight PVs were deployed to deliver home based and group sessions to twenty-one mothers experiencing perinatal depression, over a period of 6 months. Sessions were arranged in accordance to the mothers’ convenience and encouraged involvement of their significant family members especially mothers-in-law from the start of the intervention. The programme adopted the cascade model of training and supervision—the PVs were trained and supervised by non-specialist THPP facilitators, who in turn were trained and supervised by a specialist. The PVs training included a 4-day classroom and 2-day field training. The training focused on gaining counselling skills, understanding perinatal depression, using culturally appropriate pictorial illustrations to help mothers gain deeper understanding of their issues, learning behaviour activation and problem solving techniques to improve mother’s personal health, her relationship with significant others and mother–child bonding. In addition they were provided fortnightly group and field supervisions aiming to provide ongoing support and experiential learning and to ensure their motivation and fidelity to the intervention.

**Table 2:** Characteristics of the participants

*Mothers (n = 21)*	
Age	Mean = 28 years (range: 21–45 years)
Marital status	Married = 21
Education	Mean = 6.6 years (range: 0–14 years)
No. of children	Mean = 3 (range: 1–9 children)
Family structure	Joint^a^ = 17 Nuclear^a^ = 4
*PVs (n = 8)*	
Age	Mean = 33 years (range: 26–40 years)
Marital status	Married = 6 Divorced = 2
Education	Mean = 11 years (range: 10–14 years)
No of children	Mean = 2 (range: 1–4 children)
Family structure	Joint = 6 Nuclear = 2
*PHC staff (n = 5)*	
Job title	Lady health workers = 3, lady health supervisors = 1, medical officer = 1
Years of experience	Mean = 10 years (range: 5–18 years)
*Significant family members (n = 15)*	
Relationship with the mother	Husbands = 5 Mothers-in-law = 10
Years of schooling	Husbands’ mean = 8.4 years (range: 8–10 years) Mothers-in-law’s mean = 1.3 years (range: 0–8 years)

^a^Joint family structure consists of multiple generations of a family living in the same house. Nuclear family structure consists of one married couple and their children

The LHWs facilitated the identification of the potential PVs based on the person specification developed from the formative work (Table 3). As the LHWs lived and worked in the same community and had an intimate knowledge of most women in their catchment areas, they were able to recommend potentially suitable PVs to the research team. PVs were recruited following interview by the research team. The PVs were paid sustenance allowance of approximately £10 per month during the period of the study.

**Table 3:** Selection criteria for the PVs

Domain	Criteria
Qualification	At least 10 years of formal education (equivalent to GCSE)
Experience	Socio-demographic background and life experiences similar to that of the target population
	Emotional maturity/range of life experience
Personal attributes	Willingness to learn new skills
	Good interpersonal skills
	Ability to relate to mothers and their families
	Ability to maintain a balance between home and work responsibilities
	Trustworthy, empathetic, respectful and enthusiastic
Knowledge/understanding	Some understanding of mother and child health issues
Other requirements	Fluent in local language
	Able to move in the community freely including if the target population is slightly far off from her place of residence

For mothers, inclusion criteria were to be of child-bearing age (between 18 and 45), be in their third trimester of pregnancy or have a child less than 3 months old, planned to reside in the study area for 1 year, and have a score of 10 or more on the PHQ-9 [35]. Mothers with severe mental illness or serious medical conditions were excluded from the study. Participant mothers were asked to identify significant member of their family; based on their identification family members were included in the sample. Local PHC staff members including a medical officer, lady health supervisors and LHWs, who assisted the PVs in their work were included. The information sheet and the consent form was provided to all participants at least 24 h prior to the data collection, along with a verbal explanation of the study. Those who gave consent were included in the study.

### Data collection

Separate topic guides were developed for mothers, PVs, the PHC staff and mothers’ significant family members and pilot-tested. Table 4 outlines the areas included in the topic guide.

**Table 4:** Areas included in the topic guide

Mothers
Understanding of perinatal depression, its causes and treatment Understanding of the PV’s role and expectations from them Experience of receiving a peer-delivered intervention Perceived impact of the intervention Barriers and facilitators to receiving intervention
PVs
Expectations from the PV’s role Experience of receiving training and supervision Experience of working in partnership with the local PHC Experience of delivering the intervention Perceived impact of the intervention on mothers and themselves Barriers and facilitators to delivering the intervention
PHC staff
Experience of working in partnership with peer-delivered intervention programme Views about the intervention Views about the PVs’ training and supervision Perception of the PVs’ competency in delivering the intervention Perceived impact of the intervention on the mothers and the PVs
Significant family members
Understanding of perinatal depression, its causes and its treatment Understanding of the PV’s role and expectations from them Perceived impact of peer-delivered intervention Barriers and facilitators to peer volunteering

This study used both in-depth interviews and focus groups to collect data from the pilot study participants. In-depth interviews were conducted with the mothers, PVs and PHC staff. They were aimed to gain a thorough understanding of participants’ experiences, while assuring confidentiality of the information disclosed. All participants were given the choice to be interviewed either at their homes or local PHC centre. Interviews of all participants, who gave their consent, were recorded and were transcribed verbatim. For those who did not give their consent for recording, notes were taken during the interview. Focus groups were conducted with the husbands and mothers-in-law of the participant mothers. They were aimed to explore attitudes towards the PVs working in their communities and to obtain multiple perspectives on the subject matter. All recorded data were transcribed by trained transcribers.

### Data analysis

The process of data collection and analysis were carried out simultaneously. Framework Analysis approach was used to analyse data. This approach is explicitly geared towards developing policy and practice oriented findings through using systematic and transparent methods [36]. Data analysis employed all five steps of the Framework Analysis approach. Once an interview was transcribed it was read and re-read to identify emerging themes and to initiate the development of a thematic framework. Each theme and its sub-theme in the thematic framework were given an index number. This was followed by indexing, which involved systematically applying the thematic framework back to the raw data. The index number was used for identifying sections of the data and referencing it in accordance with the thematic framework. The next step called charting involved summarisation of indexed sections from the raw data and placing them on thematic charts for each set of participants. All summaries included in the chart were referenced to allow audit trail of the findings. Finally, key elements of the charts from all set of participants were critically examined to understand links and associations which facilitated the interpretation of the data.

In order to ensure the rigour of the study, a framework for applied research focusing on core principles of quality: transparency and systematicity was followed [37], along with the procedures of triangulation and reflexivity. Reflexivity was ensured through maintaining field notes and engaging in regular discussions with the research teams in Pakistan and UK. Triangulation of data sources was achieved through collecting data from different sets of participants (mothers, their husbands and mothers-in-law, PVs and PHC staff) and analysing the similarities and differences to achieve greater understanding of the topic and more confidence in the findings.

## Results

Recruitment of participants took place from January 2014 to May 2014. Data was collected through interviews (n = 34) with; mothers (n = 21), PVs (n = 8), local PHC staff (n = 5) and focus group discussions (n = 2) with; husbands (n = 5) and mothers-in-laws (n = 10). In-depth interviews were conducted at participants’ homes and lasted between 30 to 60 min. Focus groups were held at the PHC centres lasted between 75 and 90 min. The mothers and the PVs age ranged from 21 to 45 years with an average number of three and two children respectively. All were mothers apart from one PV and more than 80 % of the participants lived in a joint family system.

Three out of these four key stakeholders, the mothers, their families, and community health workers, can be conceptualized as ‘the community’ that might have a varying level of acceptability for the PVs. This level of acceptability would, in turn, be dependent on the peers’ own level of motivation for the work they are undertaking. The findings were accordingly divided into four major categories: *Facilitators* to: (a) community acceptability, and, (b) PV motivation; and *barriers* to (c) community acceptability, and, (d) PV motivation. Themes that emerged from the data within each of these categories are presented in Table 5. Quotes given in the table are illustrative.

**Table 5:** Qualitative interview results

Category	Themes	Quote (source—interviews unless stated)
1. Facilitators to community acceptability	1.1 High level of need	*I used to think a lot, whether I will survive my pregnancy or not? I was unsure and scared, everyone used to say that I did not look very well.* 08M *Women are afraid of arguments in the house. Some have very strict husbands while others have strict mothers*- *in*- *law. Women know that they have to tolerate this because if they argue they might be asked to leave home. This leaves them with no option other than to stay quiet and be compliant.* 32M
	1.2 Desirable PV characteristics	*It gave me the opportunity to off load myself. Only a mother, who has gone through similar problems, can understand how another mother is feeling.* 32M *Her nature and style of talking was really good. She was like a friend. Her company used to make me feel happy. When she used to leave, I felt someone close to me has gone.* 29M
	1.3 Linkage with local PHC system	*Nobody knows about us whereas LHWs are working for the last 18*–*19* *years. It would be really difficult for the PVs to work without their involvement.* 33PV
	1.4 Intervention perceived positive	*I think the pictures were most effective. The majority of the mothers in our village are not able to read, so just by looking at the pictures you can tell the whole story.* 02M *In every following session I used to ask her about her mood and daily routine. She used to tell me what she has done, how she has taken care of her baby. This makes me realise that she is following my suggestions. Moreover the improvement in her says it all.* 01PV
2. Facilitators to PV motivation	2.1 Personal gains	*It has given me an opportunity to learn and gain experience. I hope I can get LHW’s post, as I would like to contribute to house income and want my daughter to feel proud of me.* 07PV *Before they were sitting at home, only focusing on their children and domestic chores, now working with other women has changed their thinking for the better. It had provided them the opportunity to interact with women in their neighbourhood, which has enhanced their social functioning.* 19LHW
	2.2 Family and community endorsement	*My family is supportive, without their encouragement, I would not have done this work. It would have been really difficult to leave housework and children.* 01PV *They ask us about our programme, about our role, where we have received our training, how do we operate, what do we do? When I explain to them they get satisfied, knowing that we are doing this work with an objective.* PV05
	2.3 Good training and supervision	*I like the training…the trainers were friendly…the way they explained the content was very good. I didn’t experience any problems in understanding it. Through it I have learned a lot, which has helped me to overcome my own depression.* 15PV. *When xx (supervisor) accompanied me, mothers took me more seriously and shared their concerns more openly knowing that I have been properly trained and supervised.* IDI-15PV
3. Barriers to community acceptability	3.1 Stigma of mental illness	*She got upset when I told her that the assessment indicated that she has depression. She said that she is not mad and stopped me from coming in when I went for my next visit.* 22PV
	3.2 Societal and cultural barriers	*I could not go to attend groups; it is not a custom in Pathan families for women to visit other people’s homes unaccompanied. If I will go and my husband will find out he will get upset.* 09M *They say that she (mother) is being possessed, so instead of medicines they go for talisman (spiritual treatment).* 19LHW
4. Barriers to PV motivation	4.1 Lack of engagement of mothers	*I had to leave my housework to sit with her, so I stopped her from coming.* 13M *My husband business is not doing well, financially we are struggling, we have children to look after, we have the responsibility to marry them off and give them dowry* etc*., all these worries are pulling me down. Talking to xx (PV) can’t help me.* 09M
	4.2 Resistance from mothers family	*Groups are held outside; our women observe the veil and do not go out.* FGD-02H *Some families are financially struggling and would like to receive more than just the information given by the PV. I have the experience of upbringing five children, I can give her the information she needs. What is difficult for me is to buy fruits and medications for her, so if the PV can help with buying what is required to keep her well, it will be appreciated.* FGD-01ML

### Synthesis of the results

**Fig. 1:**
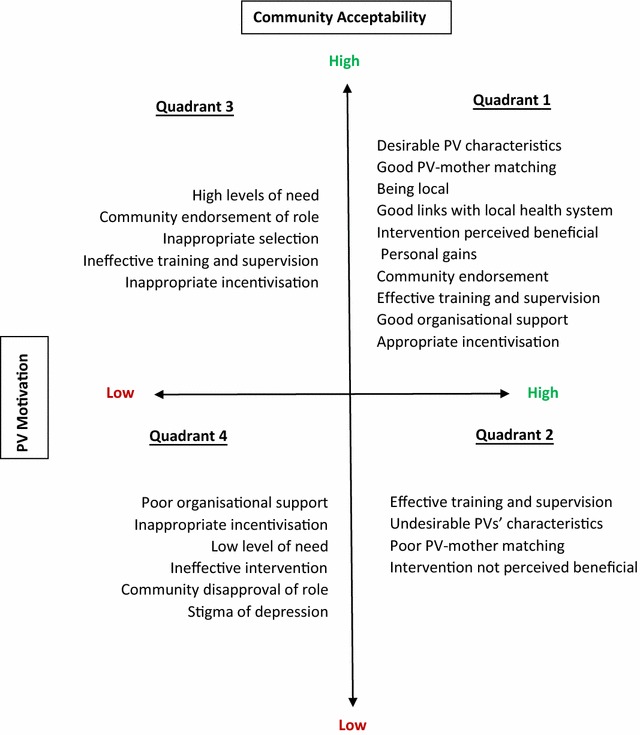
Synthesis of study findings in the form of a matrix

A synthesis of the study findings are presented in the form of a matrix (Fig. 1).

The Quadrant one (upper right) depicts an optimal peer-delivered interventional programme, where there is a high level of community acceptability and a high level of PV motivation. Our study found that the key factors contributing to the acceptability of the PVs was their desirable personal characteristics, being local and having an ability to form a trustworthy and empathetic relationship with the mother and family. Furthermore majority of the mothers found the intervention beneficial which contributed to the PVs’ acceptability. The study found PVs’ level of acceptability closely linked to their level of motivation. The PVs’ perceptions of personal gain (altruistic, opportunity or wellbeing) and endorsement from their own families and communities as key factors contributing to their motivation. PVs had good links with the local health system and were supported through adequate training and supervision. The findings indicate that the pilot programme evaluated in this study, by and large, fell within this quadrant.

The Quadrant two (lower right) depicts a programme where levels of peer motivation, their training and supervision are all good, but the programme is not acceptable to the community because the characteristics of the peers selected are not desirable, or unmatched to the community they are serving (e.g., they are perceived to be ‘foreign’ to the culture). Other key factor that could undermine the programme is the intervention perceived to be ineffective. Thus, it is possible to have motivated well-trained PVs but a programme that doesn’t work. Our study has indicated that the intervention was perceived to be useful by the community, which contributed to both the acceptability and motivation of the PVs.

The Quadrant three (upper left) shows that, in the absence of any service, many families would be willing to receive any type of support, regardless of the quality or motivation of its workforce. However, the sustainability of such a service would be questionable. Our study found high levels of need and good receptivity towards the intervention, and this contributed to its acceptability.

Lastly, Quadrant four (lower left) shows that a programme with poorly motivated unsupported PVs who lack any credibility in the community, combined with ineffective interventions that do not address stigma and cultural barriers would lead to programmes that are not only unacceptable, but could be harmful for the recipients. It would be unethical to implement such a programme. There was no evidence from our data to indicate this, therefore scaled-up implementation was recommended.

## Discussion

Results found that PVs with shared socio-demographic and life experiences of the mothers, were acceptable as delivery agents of the psychosocial intervention. The PVs level of acceptability was dependent upon a number of key factors, including their personal characteristics (e.g., empathy and trustworthiness), being local and linked to the health system, and the intervention perceived as beneficial. Their level of motivation was a key aspect of this role, and thus to their acceptability. Their motivation was related to perceived personal gain from the role, their community’s endorsement, and good training and supervision.

The study has several implications for programmes that might consider utilising PVs for mental health care delivery. The first key area is the need for such a service and appropriateness of the intervention being offered. A recent systematic review has highlighted that for a psychological intervention to be acceptable, emphasis has to be laid on its cultural appropriateness, contextual relevance and usefulness to the target population [38]. Interventions that are not culturally acceptable are unlikely to succeed, no matter how good the quality of the work-force delivering them. In the current study, all stakeholders found the intervention to be culturally appropriate, relevant to the mothers’ needs and helpful in improving the mothers’ mood, social skills and overall wellbeing, thus contributing to their acceptability.

Studies from similar settings in Pakistan have found lack of awareness of mental illnesses and stigma of depression as barriers to receiving help [39]. The THP addressed this barrier by promoting the intervention as targeting mother and child health rather than maternal depression [39], and the same strategy was employed in the current study. Other cultural barriers such as restrictions on women’s mobility and observance of *chilla ritual* (the 40-day confinement period after child birth) were addressed through delivery of the intervention via both home based and group sessions. They were arranged in accordance to mothers’ convenience to address time and schedule constraints due to their excessive domestic responsibilities and to encourage family involvement during delivery of the sessions. This was to help overcome any misapprehensions about the intervention and to ensure family’s approval and their support for the mothers.

Another key implication is appropriate selection of the PVs through matching with the target population on relevant characteristics (such as the age, gender, socioeconomic status and language) and similar life experiences (life events, psychosocial stresses, shared experience of illness or condition). In this study, there were many similarities between the mothers and PVs in terms of their age group, locality, language, number of children, gender and family structure. Other studies have also shown that age, gender [40] and language matching [41] necessary to enhance clients’ satisfaction. Other factors that contributed to their acceptability were: being perceived trustworthy and respected members of the community; having motherhood experience; able to relate and empathise with the mothers, and befriending the mothers. This is consistent with findings from other studies in which peers offered friendship, shared personal experience [42], and used empathic skills to build rapport [28, 43, 44] and were perceived respected members of the community [45].

The third implication for implementation is developing partnership models for PVs with the local health system. In the current study, the PVs’ partnership with the PHC system proved beneficial for both parties; it provided the PVs’ with an identity and enhanced the credibility of their role, and gave PHC ownership of the programme and facilitated task sharing. In the absence of such a partnership, a peer-mentoring programme in the UK found PVs experiencing problems in engaging the mothers despite being local [42]. Evidence from other studies have also indicated similar advantages of adopting a collaborative model [46] and the significance of synergy between their goals to strengthen the partnership [47]. The LHWs’ valued the work of the PVs because of its relevance to their mother and child health agendas, and felt it fulfilled a gap in the service.

Another important implication is the importance of training and supervisory support. These are crucial to ensure PVs have the adequate skills and continuous motivation, which is essential for any programme. The significance of training and supervision was also highlighted in other studies as crucial to maintain community health volunteers’ interest and motivation [48], to enhance their credibility [49] and for the success of the peer support role [28]. In this study, while most PVs found the training sufficient to prepare them for the volunteering role, some felt that a longer and comprehensive training would have equipped them better to deal with the diverse health issues of their target population. However, the PVs’ found supervisory support adequate in enhancing their skills and in dealing with work related issues and challenges. For example, one of the challenges that the PVs experienced was some mothers’ reluctance to disclose personal information, especially when both the PV and the mother belonged to the same village. However, the advantages of being local and trustworthy far outweighed the reluctance shown by some women to disclose personal information. In supervision and training, PVs were advised to focus on behaviour activation rather than interpersonal issues in women who were not comfortable discussing them. Mechanic and Meyer also found mental health patients more concerned about confidentiality issues as compared to patients with physical health problems [50]. This is especially true in closely-knit rural communities [34, 51]. In the current study field supervision, during which the supervisors accompanied the PVs in the field, enhanced the PVs community credibility and their perception of being trustworthy. Other challenges were linked to the families’ lack of awareness of psychosocial interventions, ambiguity about the PV’s role and lack of monitory incentive. The families felt that female elders of the families were best to educate mothers on maternity and child-rearing practices and PV was not required unless her input is supplemented by monetary or other tangible incentives such as medicines or dietary supplements. Such challenges could be demotivating for the PVs and needed to be addressed during supervision. According to Daniels et al., the emotional support provided through supervision was particularly helpful in settings where lay workers had not previously worked outside of their own homes [52].

The final implication for implementation is the PVs’ sustained motivation to perform well in their role, which is essential for their acceptability. In the current study several factors were identified which contributed to their motivation such as their own families endorsement to their role and approval from mothers’ families. The PV ensured the approval from mothers’ families, through highlighting the agenda of optimal child care, building rapport and involving them at all stages of intervention delivery. Evidence from literature has also indicated approval and active support from the families, as critical to the level of lay health workers’ motivation [46]. Other key motivator were altruistic gains, and personal development through improvement in knowledge and skills, potentially leading to improved job opportunities, better understanding of own emotional issues and improved confidence, self-esteem and social status. Other studies have also indicated similar factors as the driving force behind volunteering. Factors such as willingness to help others [53–56], increase in social and personal knowledge [49, 53], better job prospects [25] and improved overall wellbeing [28] are the common motivators reported by the peers in several studies. Peers, particularly from LMICs such as India, South Africa and Bangladesh found the social aspect of volunteerism as one of motivators [49, 57]. While studies on peer support in adult mental health services in HICs have indicated monetary benefit as a significant factor for peers’ motivation [28] Such findings highlight the significance of giving careful attention to contextual differences when peer-delivered programmes are being considered for implementation—this applies especially to the concept of volunteerism—a ‘one size fit all’ approach may be inappropriate for diverse contexts [34, 48].

The above implications for programmes planning to utilise peers or equivalent human resource are presented in the form of a checklist (Table 6). This checklist has six key areas, consisting of a further 21 factors which are important to gauge the acceptability and motivation of potential peers. It may be useful for programme administrators to reflect on each area and factor prior to implementation.

**Table 6:** Checklist for acceptance and motivation of peer-volunteers (CHAMPs)

*Area 1*	*Appropriate intervention*	*Yes/no*
1	Is the intervention perceived to be useful by the target population?	
2	Has the intervention been adapted for delivery by the PVs?	
3	Has the intervention been adapted for cultural relevance?	
4	Does the intervention allow flexibility of delivery (e.g., format, location)?	
*Area 2*	*Appropriate peer selection*	
5	Is there a sufficiently large pool of potential PVs willing to work?	
6	Are the PVs locally based?	
7	Are the PVs matched to the target population? (gender, age, socioeconomic status, language etc.)	
8	Do the PVs share life-experiences with the target population? (similar life events, psychosocial stresses, shared experience of illness or condition etc.)	
9	Do the PVs enjoy the trust and goodwill of the target population?	
*Area 3*	*Appropriate links with local health system*	
10	Is the PV programme supported by local health providers?	
11	Are local health providers involved in the identification, recruitment, training and embedding of PVs in the community?	
12	Are local health providers kept up-to-date with progress of the programme?	
*Area 4*	*Appropriate training and supervision*	
13	Is the training and supervision appropriate to the level of PVs qualifications and capacity?	
14	Do the trainers and supervisors possess good knowledge of local culture?	
15	Is supervision adequate to the individual needs of the PVs?	
16	Does supervision facilitate group and experiential learning?	
*Area 5*	*Appropriate organisational support*	
17	Is there an organisational structure in place to support the day-to-day work of the PV?	
18	Is the organisational structure responsive to new challenges? (e.g., emotional need, managing expectations of PVs and community)	
*Area 6*	*Appropriate incentivisation*	
19	Are PVs’ incentives matched to their expectations? [e.g., fixed monetary incentives (salary), other monetary incentives (travel, allowances), opportunities for personal development, appreciation from the community]	
20	Is the community approving of the PVs’ role?	
21	Are PVs’ own families supportive of their role?	

*This checklist is designed to help implementers assess if their peer*-*delivered programme takes into account factors that are likely to improve the community acceptance and motivation of peer*-*volunteers in their role. Any ‘No’ response should be discussed as a potential barrier to programme implementation*

The study applied robust qualitative research methodology. Framework Analysis allowed for a systematic, transparent and thorough analysis of the data. In addition, reflexivity was ensured to maintain the author’s objectivity during data collection and data analysis process. Data was collected from all participants of the pilot study, representing a range of ages and experiences, which increased the chances of establishing a realistic picture of participant experiences. The participants of the pilot study were recruited from the local community, and therefore the sample was representative of the rural population of Pakistan.

A major limitation was that it was a short-term study covering a period of 6 months in which the PVs were deployed in this role. Therefore the long-term consequences of volunteering on the PVs acceptability and motivation could not be explored. Furthermore the study was conducted in one specific rural area of Pakistan. Therefore, the findings need to be generalised with caution to other settings. Social desirability might have influenced the responses of the participants. Additionally, the PVs’ aspirations for continued community work could have resulted in them painting a more positive picture of their experience to give a good impression of their work.

Further research in this area could throw light on some unanswered questions. Future research programmes could explore the differences in peer acceptability in varying contexts. Future qualitative studies of peer-delivered programmes could consider exploring the long-term impact of peer volunteering. Another area of research could be exploring how PVs have progressed over time in terms of their personal development, empowerment and career progression, and how this might contribute to the women development agenda in low-income conservative settings.

## Conclusion

Qualitative data from a rural area of Pakistan showed that PVs were acceptable as delivery agents of a psychosocial intervention for perinatal depression to all the stakeholders. The PVs’ personal attributes such as being local, approachable, empathic, trustworthy, enjoying a good reputation and having similar experiences of motherhood contributed to their acceptability. Factors such as perceived usefulness and cultural appropriateness of the intervention and linkage with the PHC system was found vital to their legitimacy and credibility. The PVs’ level of motivation was a key aspect of this role and thus their acceptability. Factors influencing their motivation were effective training and supervision, perception of personal gain from the programme, and endorsement from their families and the community. Likely barriers to their work were women’s lack of autonomy, cultural beliefs around the perinatal period, stigma of depression, lack of some mothers’ engagement and resistance from some families. The findings are supported by studies conducted in other LMICs that have used peers as delivery agents for maternal health conditions.

Thus, it can be concluded that PVs are a potential human resource for delivery of a psychosocial intervention for perinatal depression in this rural area of Pakistan. However, programmes intending to use PVs as delivery agents for psychosocial interventions should be cognisant of six areas encompassing 21 key factors that could greatly facilitate the acceptability of PVs in such programmes.

We hope the study findings will be of interest to health planners and implementers working in settings where shortage of human resource is a barrier to mental health care, and peer-delivered interventions are being considered.

## Abbreviations

PV: peer volunteer
LMIC: low and middle income countries
THP: Thinking Healthy Programme
THPP: Thinking Healthy Programme peer delivered
PHC: primary health care
